# The ‘Sandwich dressing technique’ for management of the palate following soft tissue augmentation procedures: a case series

**DOI:** 10.1093/jscr/rjaf1013

**Published:** 2025-12-23

**Authors:** Matthew K Morris, Aditya B Patel, Chris R Cameron, Anjali Y Bhagirath

**Affiliations:** Division of Periodontics, Faculty of Dentistry, Department of Dental Clinical Sciences, Dalhousie University, 5981 University Avenue, Halifax, NS B3H 1W2, Canada; Ocean Periodontal, 1Y6, 1000-5991 Spring Garden Rd, Halifax, NS B3H 4R7, Canada; Division of Periodontics, Faculty of Dentistry, Department of Dental Clinical Sciences, Dalhousie University, 5981 University Avenue, Halifax, NS B3H 1W2, Canada; Ocean Periodontal, 1Y6, 1000-5991 Spring Garden Rd, Halifax, NS B3H 4R7, Canada; Ocean Periodontal, 1Y6, 1000-5991 Spring Garden Rd, Halifax, NS B3H 4R7, Canada; Division of Periodontics, Faculty of Dentistry, Department of Dental Clinical Sciences, Dalhousie University, 5981 University Avenue, Halifax, NS B3H 1W2, Canada; Department of Microbiology & Immunology, Dalhousie University, Halifax, NS, Canada; Ocean Periodontal, 1Y6, 1000-5991 Spring Garden Rd, Halifax, NS B3H 4R7, Canada

**Keywords:** palate, donor site, periodontal dressings, free gingival graft, connective tissue graft, soft tissue augmentation, patient comfort, postoperative pain

## Abstract

Patient comfort after soft tissue augmentation procedures (STAP), most commonly free gingival grafts (FGGs) and connective tissue grafts (CTGs), is largely determined by morbidity at the palatal donor site. Pain, bleeding, and swelling at this site are the most frequently reported causes of dissatisfaction and may limit willingness to undergo future STAP. There is a need for simple, reproducible methods that improve donor-site comfort without adding operative complexity or cost. The ‘sandwich dressing technique’ is a straightforward, reproducible palatal donor-site dressing technique designed to enhance hemostasis and comfort following STAP. Across the series, the sandwich dressing technique was feasible to implement, required no additional specialized materials, and added minimal chairside time. Patients reported acceptable comfort and manageable postoperative symptoms, with no unexpected adverse events or dressing-related complications. Within this case series, the sandwich dressing technique is a practical, easily adopted approach for palatal donor-site management after STAP.

## Introduction

Since Friedman’s 1957 description of soft tissue augmentation procedures (STAP) to increase the zone of attached gingiva, release frena/muscle pull, and deepen the vestibule, techniques have evolved substantially [1]. The most commonly performed approaches are the apically positioned flap with free gingival graft (FGG) and the coronally advanced flap with connective tissue graft (CAF + CTG) [2]. Patient-reported outcomes (PROs) following STAP are increasingly emphasized, with reports of reduced dentin hypersensitivity after root coverage [3] and improved comfort during oral hygiene around implants [4].

Despite technical advances, a recent consensus centered on PROs continues to identify autogenous grafting—most frequently harvested from the palate and, less commonly, the tuberosity—as the reference standard for phenotype augmentation [5]. However, donor-site morbidity at the palate, particularly pain, and bleeding associated with an open wound, remains a frequent source of dissatisfaction [6]. Because harvesting approaches for FGG and CTG are both palatal (the CTG is simply de-epithelialized prior to placement), the potential for palatal morbidity is shared across these procedures [7].

Multiple techniques for managing the palate have been reported in the literature, which include the following:

- Sutures with either a collagen matrix (S + CM), a collagen plug (S + CP), oxidized regenerated cellulose (S + ORC) or platelet rich fibrin (S + PRF);
- Cyanoacrylate (Cy) with or without a collagen plug (CP);
- Palatal stent (PS);
- Periodontal dressing (PD) [8].

In a randomized controlled trial of 72 patients comparing S + CP (control) to Cy + CP, S + PRF, and PS, all test groups showed improved PROs, including lower analgesic consumption, reduced pain perception, and greater willingness for retreatment; the PS group trended best among tests though differences were not statistically significant [9]. Given cost, chairside logistics, and intolerance in patients with a gag reflex, we developed a layered approach combining Cy–PD–Cy, termed the ‘sandwich dressing technique.’ The aim of this case series is to document the technique as a practical palatal management option that supports healing and patient comfort, potentially improving willingness to undergo future STAP when indicated.

## Case series

This case series is in line with the PROCESS 2020 [10] and CAse REport guidelines [11].

### Patient case selection

**Figure 1:**
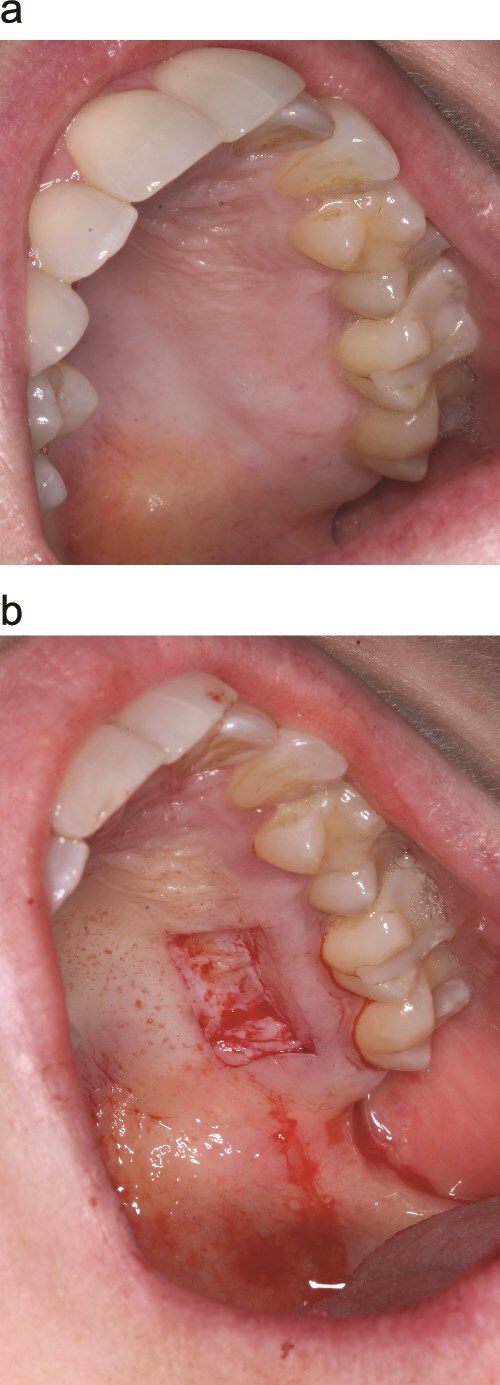
Palatal harvest site (a) palatal wound immediately after graft removal (b).

Consecutive patients indicated for STAP who had not previously undergone a STAP but were treatment-planned for potential staged procedures were invited and provided written informed consent. Palatal grafts were harvested with dimensions <20 mm in length and < 2 mm in thickness (Fig. 1a and b). The first 12 patients receiving STAP in three private periodontal practices were managed with the sandwich dressing technique. All procedures were performed by a periodontist on patients with ASA 1 or 2 and 2 patients had comorbidities.

### Surgical procedure (palatal donor-site management)

**Figure 2:**
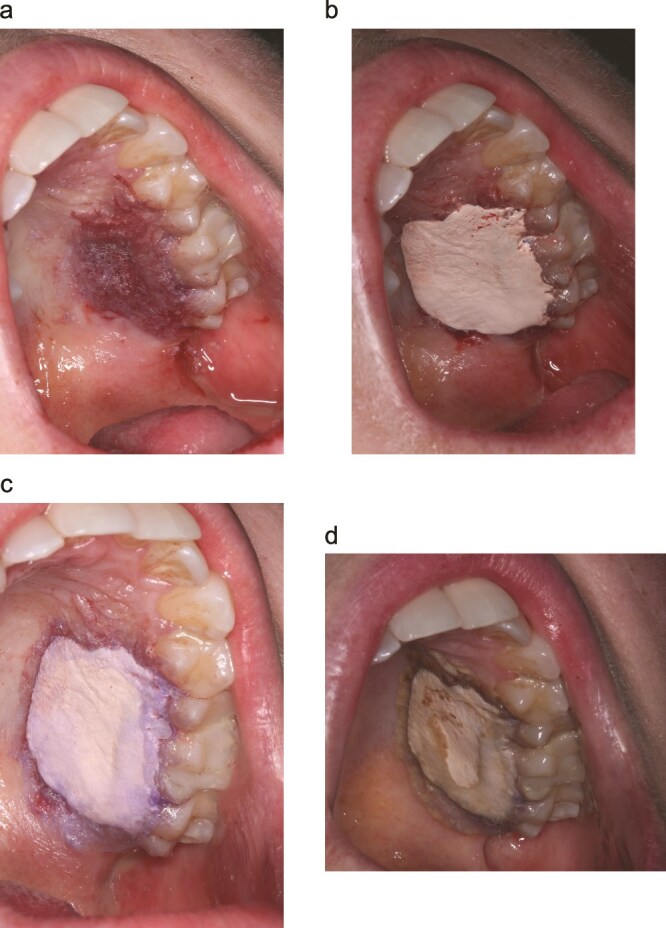
Sandwich dressing technique for palatal donor-site management. (a) First cyanoacrylate (cy) layer extended into interdental embrasures forms a stable margin. (b) Periodontal dressing (PD) adapted with firm digital pressure. (c) Second cy layer seals PD periphery to palatal mucosa creating a low-profile barrier. (d) Palatal appearance on day 14 at the post-operative appointment.

Immediately after graft harvest, the sandwich dressing technique was applied as follows:

First layer (Cy): A thin, continuous film of cyanoacrylate was applied to the palatal wound and gently extended into the adjacent interdental embrasures to create stable margins (Fig. 2a).

Core layer (PD): A periodontal dressing (Coe-Pak™) was molded to cover the harvest site and extended into the embrasures. Firm digital pressure was applied to adapt and compress the PD over the initial Cy layer (Fig. 2b).

Seal layer (Cy): A second layer of cyanoacrylate was applied over the PD, focusing on the peripheral interface to smooth the transition from PD to palatal mucosa and to seal the margins (Fig. 2c).

The appearance and sequence (Cy–PD–Cy) resemble a ‘sandwich,’ hence the name.

### Postoperative care and follow-up

Patients returned at day 7–14 for review (Fig. 2d). No patients smoked, and no dropouts occurred. At review, patients were queried regarding palatal pain, bleeding, swelling, unscheduled visits/contacts, and willingness to undergo future procedures; findings are summarized in Table 1**.**

**Table 1:** Patient characteristics and donor-site outcomes with the ‘Sandwich dressing technique’ to manage the palate following a STAP.

Characteristics	‘Sandwich dressing technique’
Sample size	12
Age range in years (mean)	26–65 (42.3)
Patients sex identified as Male: Female sex	5: 7
Number of systemically healthy patients (%)	4 (33.3%)
Number of smokers (%)	2 (16.7%)
Number of patients with diabetes (Type 2) (%)	1 (8.3%)
Number of patients with hypertension (%)	3 (25%)
Number of patients with ADHD (%)	1 (8.3%)
Number of patients with hypercholesterolemia (%)	2 (16.7%)
Number of patients with anxiety/depression (%)	2 (16.7%)
Mean number of days analgesia was taken for	2.1
Mean number of days that the ‘sandwich dressing technique’ was retentive and protected the palate	12.9
Number of patients with post-op bleeding, unscheduled visits/calls, infection (%)	0
Percentage of patients willing to undergo another STAP (%)	100

## Discussion

Predictable palatal donor-site management is central to the patient experience after soft tissue augmentation around teeth and implants.

In this series, a simple Cy–PD–Cy (‘sandwich’) layering created an immediate, low-profile seal over the harvest site that (i) stabilized the wound, (ii) reduced surface irritation from speech, eating, and tongue contact, and (iii) improved hemostasis by sealing capillary ooze.

These mechanisms plausibly explain the favorable patient-reported outcomes: no bleeding or swelling episodes, analgesic use largely attributable to the recipient site, and universal willingness to undergo future STAP. The dressing remained in place for a mean of 12.9 days (range 10–14 days)—a clinically ideal window that spans early epithelialization and minimizes daily wound care.

Compared with a palatal stent, the sandwich approach is chairside, inexpensive, and gag-reflex friendly, avoids laboratory steps, and does not require repeated insertion/removal. Relative to biologic adjuncts (e.g. PRF, collagen matrices), it uses widely available materials and adds minutes, not visits to the workflow chairside. For staged phenotype augmentation or when multiple donor sites may be needed over time, these practical advantages can lower barriers to retreatment and shorten time away from work or routine activities.

### Clinical pearls and indications

The technique is useful when [1] patients cannot tolerate a stent, [2] cost needs to be minimized, [3] thin palatal tissues make mechanical protection desirable, and [4] rapid return to normal function is a priority. Key steps are embrasure extension of the first Cy layer, firm digital compression of the PD, and peripheral sealing with the final Cy layer to prevent edge lift.

## Conclusion

The *sandwich dressing technique* is a practical, low-cost method for *palatal donor-site management* after STAP. In this series it was *well retained (~13 days)* and associated with *uneventful healing, minimal donor-site symptoms, and high willingness for future treatment*. Given its simplicity and accessibility, the technique is a reasonable option when stents are undesirable, or resources are limited; controlled trials are needed to confirm comparative benefits.
